# The Multiple Lenses on the Community Health System: Implications for Policy, Practice and Research

**DOI:** 10.34172/ijhpm.2021.73

**Published:** 2021-07-07

**Authors:** Helen Schneider, Jill Olivier, Marsha Orgill, Leanne Brady, Eleanor Whyle, Joseph Zulu, Miguel San Sebastian, Asha George, The Chaminuka Collective

**Affiliations:** ^1^SAMRC Health Services to Systems Research Unit, School of Public Health, University of the Western Cape, Cape Town, South Africa.; ^2^School of Public Health & Family Medicine, University of Cape Town, Cape Town, South Africa.; ^3^School of Public Health, University of Zambia, Lusaka, Zambia.; ^4^Department of Epidemiology and Global Health, Umeå University, Umeå, Sweden.; ^5^School of Public Health, University of the Western Cape, Cape Town, South Africa.

**Keywords:** Community Health System, Community Health, Community Health Workers, Inter-disciplinary, Collective Action, Critical Lens

## Abstract

Community health systems (CHSs) have historically been approached from multiple perspectives, with different purposes and methodological and disciplinary orientations. The terrain is, on the one hand, vast and diverse. On the other hand, under the banner of universal health coverage (UHC) and the Sustainable Development Goals (SDGs), a streamlined version of ‘community health’ is increasingly being consolidated in global health and donor communities. With the view to informing debate and practice, this paper seeks to synthesise approaches to the CHS into a set of ‘lenses,’ drawing on the collective and multi-disciplinary knowledge (both formal and experiential) of the authors, a collaborative network of 23 researchers from seven institutions across six countries (spanning low, middle and high income). With a common view of the CHS as a complex adaptive system, we propose four key lenses, referred to as *programmatic, relational, collective action* and *critical lenses*. The lenses represent different positionalities in community health, encompassing macro-level policy-maker, front-line and community vantage points, and purposes ranging from social justice to instrumental goals. We define and describe the main elements of each lens and their implications for thinking about policy, practice and research. Distilling a set of key lenses offers a way to make sense of a complex terrain, but also counters what may emerge as a dominant, single narrative on the CHS in global health. By making explicit and bringing together different lenses on the CHS, the limits and possibilities of each may be better appreciated, while promoting integrative, systems thinking in policy, practice and research.

## Background

The idea of ‘Community Health,’ while by no means recent, is gaining traction as part of new global agendas on primary healthcare (PHC) and universal health coverage (UHC). This (re)emerging idea brings together various strands of thinking and programming that have evolved over the past two decades. These include renewed support for community health worker (CHW) programmes as part of the Millennium (and now Sustainable) Development Goals (SDGs)^1^; ‘community system strengthening’ linked to the Global Fund for HIV/AIDS, Malaria and TB^2^; and a sustained interest in the tools of social accountability (eg, citizen score cards or health facility committees) as engines of health system change.^3^ Awareness of the role community engagement and mobilisation played in global and humanitarian crises – such as the Ebola epidemic in West Africa – has also contributed to elevating the status of ‘community’ in health systems.^4^ Community-based strategies will in all likelihood emerge as a key element of the responses to the coronavirus disease 2019 (COVID-19) pandemic.

There have been multiple global initiatives launched in response to this increased interest in community health. For example, in 2017, a coalition of bilateral, multi-lateral and international non-governmental organisations held a conference entitled ‘Institutionalising Community Health’ in Johannesburg, South Africa. A ‘Community Health Roadmap,’ led initially by the United States Agency for International Development (USAID), United Nations International Children’s Emergency Fund (UNICEF) and philanthropic foundations (https://www.communityhealthroadmap.org/), but increasingly involving an array of other global actors (including the World Health Organization [WHO]), was subsequently launched in September 2019 as a follow-up to the conference. The Roadmap seeks to consolidate the activities and direct the investments of these international agencies in an initial group of 15 low- and middle-income ‘RoadMap’ countries. In these countries, an integrated approach to supporting country-led priorities for community health, aimed at ‘bridging the gap’ on healthcare access and outcomes, particularly for women and children, is being developed. The RoadMap process envisages community health plans drawn up by national governments ‘and their key partners’ with cross country support from a RoadMap secretariat on six priorities – financing; reducing fragmentation of donor initiatives (eg, in supply chains); optimisation of current strategies (eg, through improved quality); future fit (further expansion); performance management (including monitoring); and generating high level commitment. These global processes are associated with an array of consensus statements, guidelines, web portals, e-health technologies, investment cases, toolkits and research prioritisation exercises on community health, that collectively constitute a veritable ‘epistemic’ project^5^ to shape global thinking and policy direction at country level.

Given the fragmented, inefficient and poorly governed terrain of mostly disease-specific community interventions in many countries,^6^ commitment on the part of donors towards more holistic approaches and greater synergy under the banner of UHC and the SDGs may be beneficial. For example, in Malawi, a country with long-standing community health initiatives, the National Community Health Strategy lists 19 ‘development partners’ (five ‘funders’ and 14 ‘implementing partners’) (https://www.communityhealthroadmap.org/malawi). Harmonising approaches and mobilising additional investments could reduce transaction costs and allow country players to more effectively steer their community health systems (CHSs). Yet, these developments also raise important questions, especially when seen from the vantage points of national and local actors involved in designing, supporting and researching CHSs. How will this new global consensus, backed by dollars, influence decision-making at national and sub-national levels, where community level strategies have evolved in context-specific ways over decades^7^? What assumptions regarding health-related assets, action and agency in communities are being put forward, when these are often observed to emerge in locally contingent, socially determined and complex ways, addressing a multiplicity of needs and goals^8,9^? What does renewed interest in intervening in community health represent, in the context of UHC and a global economic and political order vastly different to the era of the Alma Ata Declaration when global agendas for community health were first set? What systems of global governance are being put into place and what forms of knowledge generation and evidence are being prioritised in these systems^10^?

Answering these questions requires recognising the existence of multiple perspectives on the CHS. The answers have implications for approaches to policies and programmes in community health, to community responsiveness and participation and to research in the field. There is the danger that in codifying community health under the banner of UHC, interventions become type cast in a menu of strategies (eg, mhealth or score cards) and instrumental goals (eg, reduced mortality), with little space for considerations of social complexity, diversity, context-specificity and power. In 2018, a number of players called for a multi-disciplinary agenda in the field of community health, with less ‘hubris’ and more ‘humility and humanity,’ both with respect to action and what counts as good evidence.^9^

Taking our vantage point as country-level researchers of community health, concerned with system strengthening while also mindful of global developments, this paper seeks to synthesize approaches to the CHS in a set of lenses and their implications for research, policy and practice. We present the lenses as analytically distinct, recognising that in practice, actors and programmes may hold more than one lens simultaneously or sequentially over time, even if elements of the different lenses may be contradictory or in competition. National policy-makers, local implementors, community members and human rights advocates may all approach the CHS in their own way and with different primary goals – whether related to health outcomes, greater responsiveness, co-production, empowerment or community mobilisation. In their review of community engagement in health, Brunton and colleagues^11^ categorise these purposes into two broad ‘inclinations’ – a utilitarian health system perspective and a social justice perspective – along which the range of purposes and positions can be mapped.

Our use of the term CHS is deliberate, which we define as “the set of local actors, relationships, and processes engaged in producing, advocating for, and supporting health in communities and households outside of, but existing in relationship to, formal health structures.”^12^ The CHS is embedded within other systems - both the wider community system and the formal health system, and is also complex and adaptive, capable of emergence and self-organisation. As implied by the definition, ‘community’ refers to place (geography) as well as shared identity and networks (interests), that may extend beyond the boundaries of a locality.^11,13^ As with emerging definitions of ‘community health,’ the notion of the CHS is wider than a single form of provision (eg, CHW programmes), but more specific than the general idea of ‘community’ as the ‘demand’ side of the health system as a whole.^14^

 In the sections which follow, we describe the four lenses, giving examples of each and consider the implications of the lens for policy, practice and research. We discuss synergies and tensions between lenses and conclude on the importance of retaining multiple perspectives in advancing the potential of the CHS as a sphere of meaningful action in health systems.

## Methods

The four lenses were arrived at by a network of 23 public health researchers and implementers from seven institutions across six countries **(**Zambia, South Africa, Sweden, Tanzania, Uganda and Guatemala) who have been collaborating and meeting regularly on the theme of CHSs. This collective brings together a range of disciplinary backgrounds and research interests from across income (low, middle and high) settings. In June 2019, these collaborators held a one-week workshop in Lusaka, Zambia, to formulate, in a systematic manner, a research agenda for CHSs. In preparation for this workshop, participants were asked to draw on their years of tacit, experiential and empirical knowledge to define their disciplinary perspective and propose key themes of action and research on the CHS. The key themes of action were grouped into an initial set of lenses, presented at the start of the workshop, and through various forms of representation (such as rich pictures and text) these lenses were defined and elaborated, including the research methodologies typical to each (see workshop report^15^). The final version of the lenses was arrived at in a follow-up workshop held in March 2020. The lenses are thus not derived from theory or literature, but through consensus in a multi-disciplinary group of public health and health system researchers and implementers. However, they are not dissimilar to the typology of approaches to community mobilisation and engagement proposed by others.^11,16^ We also do not claim to present a complete set of lenses, but rather those that reflect our orientations as embedded researchers in the policy and practice environments of particular health system contexts. We regard each lens and associated practices and research methodologies as equally valid and legitimate, even if some may be more recognised and developed than others.

## Results

 We briefly describe the process from identification of key themes to codification into lenses, and then elaborate on the four lenses adopted and implications for policy, practice and research.

###  From Themes of Action to Key Lenses

When participants were asked to propose themes on the CHS to explore at the workshop, they offered a range of seemingly disparate responses. Reflecting their varied interests and backgrounds, some focused on the role of CHWs, others on citizen mobilisation and advocacy; some on specific programmatic areas (eg, adolescent reproductive health), others on health system building blocks (eg, human resources and financing); some on health sectors, others on multi-sectoral development; and some on macro-level policy and design, others on frontline action and implementation. These differences encouraged us to acknowledge and more carefully consider our starting points and assumptions on the CHS, which we then framed as ‘lenses.’ The lenses reference ways of seeing and different vantage points (outside and inside) on the CHS, and were initially categorised as ‘hardware,’ ‘software,’ ‘responsiveness’ and ‘critical perspectives.’^15^ Through discussion these evolved into a set of four lenses – referred to as *programmatic, relational, collective action* and *critical lenses* – which represent a combination of different positionalities and purposes in CHSs.

###  The Four Lenses Defined

Box 1 defines each of the lenses with respect to positionality and purpose.

**Box 1. Key Lenses on the Community Health System**

- The ‘programmatic’ lens involves looking ‘into CHS’ as the site of formal programming, most commonly in CHW programmes, but also other outreach activities of the formal health system, such as adherence clubs, women’s groups and clinic committees. This lens is typically associated with national ministries of health and has a utilitarian purpose such as improved access or reduced mortality.
- The ‘relational’ lens, as with the programmatic lens, has an instrumental health system purpose but the emphasis shifts from the what (design) to the how (implementation) of programmes. It typically speaks to frontline realities at the interface of the CHS and the formal health system in a bottom-up fashion. In this lens, purposes may encompass a range of intermediate goals (such as empowerment), through forms of co-production or co-creation.
- The ‘collective action’ lens takes the perspectives, priorities and actions of communities (rather than the health system) as starting points. Actors – as individuals or groups – establish their own agendas, form networks and act collectively on health, independent of programmes and mechanisms of the formal health system. Although the purposes in this lens are heterogenous they often have a rights focus, including seeking greater responsiveness from the formal health system.
- The ‘critical’ lens takes a political-economy perspective and examines dominant discourses on the CHS. It asks, for example: What interests lie behind current global developments? What would a decolonial perspective on the CHS entail? How do we ensure that contextually relevant programmes emerge at national and sub-national levels? By surfacing the often invisible societal forces which shape policy and practice at macro, meso and micro levels, this lens ultimately has a social justice purpose.

Abbreviations: CHS, community health system; CHW, community health worker.

###  The Programmatic Lens

A programmatic lens starts from the premise of the CHS as a bounded geographical space, consisting of a defined set of actors, populations and programmes, established through national policies and plans with measurable goals. The CHS forms part of a continuum with the PHC system that provides support and referral for the CHS. This lens is reflected in a growing number of national community health strategies^17-19^ and international guidance on community health. For example, USAID’s Community Health Framework^20^ proposes an ‘eco-system’ of health specific (household and community providers) and health enabling (families and communities) components, engaging individuals within communities in order to achieve specific targets (eg, coverage, mortality).

The Community Health Roadmap (https://www.communityhealthroadmap.org/) lists a variety of functions under the ambit of the CHS. These include not just CHW programmes, the traditional focus of CHS’s,^12^ but all community-based health promotion and service delivery initiatives through the range of community organisations and multi-sectoral action. Malawi’s Community Health Strategy (2017-2022) provides an example of this new wave of thinking.^21^ The Strategy’s vision is ‘improved livelihoods’ with reduced maternal and child mortality as immediate goals, to be achieved through an integrated, team-based approach to all community-based health activities. These include the well-established CHW (Health Surveillance Assistant) programme, a variety of volunteers linked to disease-specific interventions, and the structures of community participation such as Village Health Committees and Health Centre Advisory Committees. These will be supported by new systems (information, supply chains, district support units) and tools and technologies (such as citizen score cards or mhealth).

In this lens on the CHS, the priorities for action are to establish designs, structures and strategies that are evidence-informed, affordable and integrated, in a continuum with the PHC system. In addition to implementing community-based interventions addressing key disease burdens, this lens would typically be concerned with the building blocks of the CHS – human resource systems (scope, selection, training, supervision etc of community-based cadres), financing, technologies (such as rapid diagnostic tests), planning cycles, and governance arrangements that ensure coordinated action. The core assumptions are rationality and linearity in decision-making and implementation, with performance shaped through targets and standardised approaches. Communities are assumed to be (more-or-less) homogenous, amenable and responsive to programmes and inherently pro-social. Knowledge generation (research) in this lens would favour well-controlled experiments of technical interventions, programme and performance evaluations, implementation research, cost-effectiveness studies and investment cases.^10^

###  The Relational Lens

In contrast to the programmatic lens, the relational lens views the CHS as a ‘peopled’ system of relationships – formal or informal – involving a wide variety of actors, interests and expressions of power that together constitute a social system. The CHS has all the hallmarks of an open, complex adaptive system, consisting of multiple interactions and feedback loops resulting in non-linear and unpredictable effects, emergent phenomena and self-organisation.^22^ Relationships in the CHS are shaped by these complex interactions, embedded in histories, resources, social hierarchies, cultures and norms of communities creating unique contexts in which programmes and interventions ‘evolve dynamically.’^9,13^ Contrary to the programmatic lens – this lens thus views communities as diverse and dynamic rather than homogenous.

Communities are also not passive recipients of programmes handed down through hierarchies. Actors at all levels have the capacity to express agency and do so in multiple ways – actively adopting, selectively implementing, reshaping or resisting programmes in order to fit their everyday realities and routines. If these factors are not taken into account, well-intentioned policies and programmes may not be adopted at all or have negative consequences. They may exacerbate damaging social inequalities in communities, such as caste and gender relations in India^23^ or deepen conflict and competition between frontline actors. Poor relationships between community-based providers and the PHC system, underpinned by hierarchies of status and power and impacting on performance, have been described in many settings.^23-27^ Policies may fail to gain legitimacy or become stripped of their original intent when implemented. A recent study in Malawi highlighted the scepticism of frontline players towards the new accountability arrangements in the country’s Community Health Strategy,^19^ while in South Africa a new CHW policy was reconfigured at multiple interfaces as it was implemented, ultimately leading to a considerable ‘thinning’ of original purpose.^24^

Seen through this lens, the CHS has potential, but is far less ‘programmable’ or amenable to ‘mobilisation’^25^ and systems of ‘social accountability’^3^ than imagined in policies and plans, which often fail to deliver as intended in the everyday reality of health systems. Here, in addition to shortcomings in resources, implementation failures are seen as being a result of programmes ignoring the inherently complex and social nature of interventions and community systems. However, despite well-documented observations of the relational and social nature of the CHS, these phenomena are ‘rarely problematized’ in official policy.^23^

A relational lens on practice in the CHS would prioritise engagement with actors in the CHS through forms of co-production, implementing context-specific approaches, tailored to local needs and realities, encouraging experimentation and learning, and nurturing social skills for navigating and negotiating relationships.^9,13^ These approaches have been successfully applied in interventions addressing health programmes (HIV, maternal-child and reproductive health and others), across a variety of settings,^22,26-28^ and were key to overcoming the Ebola epidemic in West Africa.^4^ Research in this lens would typically adopt participatory action research approaches,^27^ apply methods drawn from the field of complexity science,^22^ identify community capabilities,^13^ and analyse power^23,24^ and actor networks.^29^

###  The Collective Action Lens

This lens is primarily concerned with mechanisms and processes which enable actors in the CHS to mobilise, collaborate and act collectively on health. The lens recognises that cooperative behaviours and coordinated action are the ultimate drivers of change in the CHS, often referred to as ‘community mobilisation.’ As alluded to in the relational lens, community mobilisation can be supported but not engineered in CHS programmes and “…interventions differ critically from CHW [and other] programmes in their emphasis on community members themselves identifying, prioritising and owning solutions to local health problems.”^25^

Collective action in the CHS may emerge to meet particular social, instrumental or identity-related needs, in opposition to social injustice or in reaction to failures of the health system. Community mobilisations may be local and specific or evolve into larger movements. An example of the latter is the South African Treatment Action Campaign (TAC), formed by rights-based AIDS activists and people living with HIV in the late 1990s, which developed into a significant social movement.^30,31^ The TAC’s messaging and branding (‘HIV positive’), emphasis on treatment literacy and considerable political and networking capabilities, enabled it to swing public opinion in its favour. The TAC is widely credited for ensuring a relatively well-functioning programme of universal access to anti-retroviral therapy through South Africa’s public health system.

Forms of collective action also include the myriad of self-help, peer support groups and associations established around specific health needs such as mental ill-health^32^ or chronic non-communicable diseases, collectives of marginalised groups (eg, sex-workers),^33^ or the charitable initiatives of faith-based organizations. It is important to recognise that not all collective action on health will be in the interests of good health or promote equity. For example, community alliances (eg, religious or health professional) may form to limit access of youth to sexual and reproductive health services, or in moments of crisis create community scapegoats (eg, foreigners). Collective action, thus, has to be understood as inherently political ie, as local expressions of particular values, interests and power.

Drawing on observations of ‘endogenous’ forms of community mobilisation in Nigeria to tackle the ‘under-governance’ of local health systems, Abimbola^8^ seeks to theorise the conditions of collective action for health. He challenges the ‘a priori’ “assumption that people necessarily want to participate in decisions about their own healthcare…” recognizing that the costs (time, resources etc) involved in ‘participation’ may outweigh any benefits. This is also referred to as the ‘collective action problem,’^25^ which requires a necessary alignment of contextual, geographical and institutional factors to be overcome.^8^ Collective action is also driven by common social or political identities (eg, ‘rights activist’) and experiences of successful challenges to injustices.^34^ Small-scale, ‘improvisational’ mobilisations may engender and catalyse more significant cycles of change, while network building within and across communities enhances their ‘social capital’ and capacity for influence.^16^

Research in this lens would be focused on the various contexts, drivers and conditions of collective action outlined above, and has been approached from a number of disciplinary perspectives (economic, political, social-psychological, development).^25^

###  The Critical Lens

The critical lens locates the CHS in a wider set of social, political and economic relations that transcend national boundaries and which form the context for health sector decision-making, including in the CHS. For example, the ideas of community participation and the formalisation of CHW programmes gained favour in many countries following the Declaration of Alma Ata in 1978. These ideas formed part of expansive understandings of state-enabled bottom-up development and empowerment in post-independence societies. In the structural adjustment programmes of the 1980s and 1990s many of these programmes fell apart, with a retreat from the role of the state and comprehensive PHC.^35,36^ The Millennium Development Goals, while signalling a return of funding for the ill-health burdens of the global south and for CHW programmes in particular, entrenched a disease-specific approach to community health, promoting “neutral technical solutions, implemented in local settings, without attention to the wider economic and political inequalities that drive them.”^16^

The second decade of the 21st century has seen the consolidation of a global neo-liberal economic order and the unparalleled growth of markets and market thinking in health systems across the globe.^37^ Nowhere is this more evident that in the field of ‘global health,’ where public-private partnerships have become the norm, with corporate penetration into health sector provision and supply chains of low- and middle-incomes, including in the CHS. For example, ‘Project Last Mile,’ a partnership between Coca-Cola, USAID, The Bill and Melinda Gates Foundation and the Global Fund to Fight AIDS, Tuberculosis and Malaria, supports community drug distribution programmes in South Africa and HIV health promotion for adolescent girls in eSwatini, using Coca-Cola’s supply chains and branding.^38^ In 2018, companies at the World Economic Forum pledged US$100 million for the sale and distribution of commodities through CHWs in six countries, aiming to create “super-scalable networks of ‘Avon-like’ health entrepreneurs.”^39^ USAID has published guidance for ‘private capital’ seeking investment opportunities in ‘global health’^40^ and its Community Health Framework^20^ draws extensively in its framings on private sector involvement in community health, including in the supply of mobile phone technologies. These approaches represent strategic interests very far removed from their intended beneficiaries and even less the goal of “social change in favour of the marginalized.”^16^

A critical lens seeks to uncover the often hidden nature of these power relations through analyses of ‘ideas, interests and institutions,’^41^ drawing, for example, on political-economic frameworks,^42,43^ critiques of knowledge generation on the CHS,^44^ informed by decolonial perspectives,^45^ discourse analysis^46^ and feminist and inter-sectional theories.^47^ Gaitonde et al,^48^ in their analysis of the Indian ‘communitisation’ programme, illustrate well the roles of different circulating discourses (along the instrumental-social justice divide outlined earlier) amongst mid-level managers and how these materially shape the designs of programmes. By uncovering the wider political, economic and social relations at play, the critical perspective provides a way for marginalized groups to recognize injustices they may have internalized as ‘natural’ and to challenge these through forms of collective action.^16^ In this way, ‘grand narratives’ become linked to the everyday practice of social change within the CHS and health systems.

 The Table below summarises the different lenses on the CHS and their implications for policy, research and practice.

**Table:** Lenses on the Community Health System: Purposes, Positionalities and Implications

**Lens**	**Purpose of CHS**	**Positionality**	**Policy**	**Practice**	**Research**
Programmatic	Instrumental – delivery of services, health outcomes	Ministries of health	Fixed elements: financing, supply chains, information systems, cadres, structures	Programme and budget cycles, Indicators and reporting, Donor coordination mechanisms, Structures of decision-making	Costing and investment cases, effectiveness, M&E
Relational	Instrumental –programmes co-produced	Health system implementers	Core rules and with flexible, context specific components, feedback mechanisms with course correction	Negotiating, navigating, learning by doing	Community capability and assets, complex adaptive systems, power analyses, realist evaluations, action-research
Collective action	Justice and rights – responding to the needs and priorities as defined by communities	Actors within the CHS	Values of inclusivity, equity and participation Mechanisms of dialogue	Collaborative action, social mobilisation, activism	Social movements, emergence and self-organisation, social-psychology, collective action
Critical	Social justice – CHS as a site of social and political struggle	Actors within and beyond the CHS	The nature of the state and its key orientations (social-democratic, neo-liberal etc)	Analyses which support collective action, political struggle and policy reforms	Discourses, political economy decoloniality

Abbreviation: CHS, community health system.

## Discussion and Conclusions

 CHSs have been approached from multiple perspectives, with different purposes and methodological and disciplinary orientations. The terrain is, on the one hand, vast and diverse, while on the other hand increasingly consolidated into a streamlined version of ‘community health’ in the global health and donor community. Drawing on our collective and varied experiences as country level players engaged in supporting and researching the CHS, we have sought to synthesize and distil the different lenses on the CHS. This is important, to make sense of a complex terrain, to inform policy, practice and research agendas, and to better counter what may emerge as a dominant, single narrative on the CHS.

Starting from our common understanding of the CHS as a complex adaptive system, embedded within other systems (including the formal health system), we recognise that ‘top-down’ policies and programmes are important (the programmatic lens), but always exist in a tension with ‘bottom-up’ realities.^22^ Community interventions need to be adapted to specific local contexts, with meaningful community engagement that acknowledges relations of power both within the CHS and the wider health system (the relational lens).^9^ Action on the CHS needs to be attuned and responsive to emergent forms of community mobilisation (collective action lens), while at the same time cognisant of wider social and political forces shaping the CHS (critical lens). When brought together, the lenses can provide the basis for the integrative, systems thinking in policy and practice that is required to effectively advance equity and access through the CHS. When seen in relation to the other lenses, the limits, possibilities and inter-dependence of each lens is better appreciated, thus enabling “more humility and less hubris.”^9^ Recognition of the multiple lenses should also lead to a greater openness to inter-disciplinary research and the value of multiple research methodologies.

 Advancing a truly systems approach to the CHS requires structures and processes of governance that embrace different perspectives, provide the space and mechanisms for voicing and negotiating difference, foster learning and adaptability, and which are held together by common values such as equity and social justice. These mechanisms would ideally be part of networks of cooperation and exchange between embedded researchers, policy-makers and practitioners within and across countries.

However, simply stating the necessity for different lenses will not necessarily alter the interests behind the different approaches to the CHS. For example, if the CHS is seen as a sphere for advancing donor and commercial interests or exercise bureaucratic power,^49^ voices for social justice and rights are likely to be marginalised. Even if there is acceptance that the lenses can productively complement each other, they ultimately represent different purposes and may compete in their assumptions. For example, community mobilisation will be most effective if it can draw on the resources and support of the formal health system, but that system cannot *require* of communities that they act together. Critical perspectives uncover the deeper social and political forces which need to be addressed for meaningful change, but policy-makers and implementors need to find pathways to act in the reality of here and now, and make imperfect decisions and trade-offs. The value of recognising different lenses on the CHS can thus only be realised when it also includes consideration of decision-making structures, interests and power.

## Acknowledgements

The views presented in this article were first elaborated in a workshop report, entitled: Lenses, Metaphors and Research Priorities on Community Health Systems, held at the Chaminuka Lodge, Lusaka, June 10-14, 2019, available at https://www.uwcsoph.co.za.

## Ethical issues

 Data reported are based on a workshop process on which all the authors participated. No primary data were collected.

## Competing interests

 Authors declare that they have no competing interests.

## Authors’ contributions

 All authors contributed to the conceptualisation and elaboration of key concepts in the paper. HS led the conceptualisation and drafting of the article.

## Funding

 Swedish Foundation for International Cooperation in Research and Higher Education (STINT), the South African National Research Foundation (NRF), and a network grant (ResearchLink) from the Swedish Research Council (Vetenskapsrådet, VR).

## Authors’ affiliations

^1^SAMRC Health Services to Systems Research Unit, School of Public Health, University of the Western Cape, Cape Town, South Africa. ^2^School of Public Health & Family Medicine, University of Cape Town, Cape Town, South Africa. ^3^School of Public Health, University of Zambia, Lusaka, Zambia. ^4^Department of Epidemiology and Global Health, Umeå University, Umeå, Sweden. ^5^School of Public Health, University of the Western Cape, Cape Town, South Africa.

## Members of the Chaminuka Collective

 The Chaminuka Collective consists of the following members, displayed with their affiliations:

**Charles Michelo**, School of Public Health, University of Zambia, Lusaka, Zambia; **Chama Mulubwa**, School of Public Health, University of Zambia, Lusaka, Zambia; **Adam Silumbwe**, School of Public Health, University of Zambia, Lusaka, Zambia; **MalizganiChavula**, School of Public Health, University of Zambia, Lusaka, Zambia; **MargarateMunakampe**, School of Public Health, University of Zambia, Lusaka, Zambia; **Wanga Zulu**, School of Public Health, University of Zambia, Lusaka, Zambia; **Tumelo Assegaai**, School of Public Health, University of the Western Cape, Cape Town, South Africa; **Uta Lehmann**, School of Public Health, University of the Western Cape, Cape Town, South Africa; **SiriliNathaneal**, School of Public Health and Social Sciences, Muhimbili University, Dar es Salaam, Tanzania; **Moses Tetui**, Department of Health Policy Planning and Management, Makerere University, Kampala, Uganda; **Frida Johnsson**, Department of Epidemiology and Global Health, Umeå University, Umeå, Sweden; **Isabel Goicolea**, Department of Epidemiology and Global Health, Umeå University, Umeå, Sweden; **Anna-Karin Hurtig**, Department of Epidemiology and Global Health, Umeå University, Umeå, Sweden; **Alison Hernandez**, Centre for Health Equity and Governance in Health Systems, Guatemala City, Guatemala; **Lance Louskieter**, School of Public Health and Family Medicine, University of Cape Town, Cape Town, South Africa.

## References

[1] Maher D, Cometto G (2016). Research on community-based health workers is needed to achieve the sustainable development goals. Bull World Health Organ.

[2] Global Fund. Technical Brief: Community Systems Strengthening. Geneva, Switzerland: Global Fund; 2019.

[3] Boydell V, McMullen H, Cordero J, Steyn P, Kiare J (2019). Studying social accountability in the context of health system strengthening: innovations and considerations for future work. Health Res Policy Syst.

[4] Barker KM, Ling EJ, Fallah M (2020). Community engagement for health system resilience: evidence from Liberia’s Ebola epidemic. Health Policy Plan.

[5] Shiffman J (2014). Knowledge, moral claims and the exercise of power in global health. Int J Health Policy Manag.

[6] Tulenko K, Møgedal S, Afzal MM (2013). Community health workers for universal health-care coverage: from fragmentation to synergy. Bull World Health Organ.

[7] Bennett S, George A, Rodriguez D (2014). Policy challenges facing integrated community case management in sub-Saharan Africa. Trop Med Int Health.

[8] 8 Abimbola S (2020). Beyond positive a priori bias: reframing community engagement in LMICs. Health Promot Int.

[9] George AS, LeFevre AE, Schleiff M, Mancuso A, Sacks E, Sarriot E (2018). Hubris, humility and humanity: expanding evidence approaches for improving and sustaining community health programmes. BMJ Glob Health.

[10] Agarwal S, Kirk K, Sripad P, Bellows B, Abuya T, Warren C (2019). Setting the global research agenda for community health systems: literature and consultative review. Human Resour Health.

[11] Brunton G, Thomas J, O’Mara-Eves A, Jamal F, Oliver S, Kavanagh J (2017). Narratives of community engagement: a systematic review-derived conceptual framework for public health interventions. BMC Public Health.

[12] Schneider H, Lehmann U (2016). From community health workers to community health systems: time to widen the horizon?. Health Syst Reform.

[13] George AS, Scott K, Mehra V, Sriram V (2016). Synergies, strengths and challenges: findings on community capability from a systematic health systems research literature review. BMC Health Serv Res.

[14] Sacks E, Morrow M, Story WT (2018). Beyond the building blocks: integrating community roles into health systems frameworks to achieve health for all. BMJ Glob Health.

[15] Lenses, Metaphors and Research Priorities on Community Health Systems. Report of a workshop held at the Chaminuka Lodge, Lusaka, Zambia 10-14 June 2019. https://www.uwcsoph.co.za/.

[16] Campbell C (2014). Community mobilisation in the 21st century: updating our theory of social change?. J Health Psychol.

[17] Republic of Zambia. Community Health Strategy 2017-2021. Zambia: Ministry of Health; 2017.

[18] McCollum R, Otiso L, Mireku M (2016). Exploring perceptions of community health policy in Kenya and identifying implications for policy change. Health Policy Plan.

[19] Angwenyi V, Aantjes C, Kondowe K (2018). Moving to a strong(er) community health system: analysing the role of community health volunteers in the new national community health strategy in Malawi. BMJ Glob Health.

[20] USAID. Community Health Framework: Distilling decades of Agency experience to drive 2030 Global Goals. USAID; 2015. https://www.chwcentral.org/sites/default/files/USAID-Community-Health-Framework_Version-1-0_October-28th-2015.pdf.

[21] Republic of Malawi. National Community Health Strategy 2017-2022. Malawi: Ministry of Health; 2017.

[22] Sarriot E, Kouletio M (2015). Community health systems as complex adaptive systems: ontology and praxis lessons from an urban health experience with demonstrated sustainability. Syst Pract Action Res.

[23] Ved R, Scott K, Gupta G (2019). How are gender inequalities facing India’s one million ASHAs being addressed? policy origins and adaptations for the world’s largest all-female community health worker programme. Hum Resour Health.

[24] Assegaai T, Schneider H (2019). The supervisory relationships of community health workers in primary health care: social network analysis of ward-based outreach teams in Ngaka Modiri Molema district, South Africa. BMJ Glob Health.

[25] Mishra A (2014). ‘Trust and teamwork matter’: community health workers’ experiences in integrated service delivery in India. Glob Public Health.

[26] Zulu JM, Kinsman J, Michelo C, Hurtig AK (2014). Hope and despair: community health assistants’ experiences of working in a rural district in Zambia. Hum Resour Health.

[27] Scott K, Shanker S (2010). Tying their hands? institutional obstacles to the success of the ASHA community health worker programme in rural north India. AIDS Care.

[28] Kok MC, Namakhoma I, Nyirenda L (2016). Health surveillance assistants as intermediates between the community and health sector in Malawi: exploring how relationships influence performance. BMC Health Serv Res.

[29] Lehmann U, Gilson L (2013). Actor interfaces and practices of power in a community health worker programme: a South African study of unintended policy outcomes. Health Policy Plan.

[30] Gram L, Daruwalla N, Osrin D (2019). Understanding participation dilemmas in community mobilisation: can collective action theory help?. J Epidemiol Community Health.

[31] Lunsford SS, Fatta K, Stover KE, Shrestha R (2015). Supporting close-to-community providers through a community health system approach: case examples from Ethiopia and Tanzania. Hum Resour Health.

[32] Muhumuza Kananura R, Tetui M, Bua J (2017). Effect of a participatory multisectoral maternal and newborn intervention on birth preparedness and knowledge of maternal and newborn danger signs among women in Eastern Uganda: a quasi-experiment study. Glob Health Action.

[33] Zulu JM, Kinsman J, Hurtig AK, Michelo C, George A, Schneider H (2019). Integrating community health assistant-driven sexual and reproductive health services in the community health system in Nyimba district in Zambia: mapping key actors, points of integration, and conditions shaping the process. Reprod Health.

[34] Ahmad H (2013). The Treatment Action Campaign and the three dimensions of lawyering: reflections from the rainbow nation. SAHARA J.

[35] Heywood M (2009). South Africa’s Treatment Action Campaign: combining law and social mobilization to realize the right to health. J Hum Rights Pract.

[36] Worrall H, Schweizer R, Marks E, Yuan L, Lloyd C, Ramjan R (2018). The effectiveness of support groups: a literature review. Ment Health Soc Incl.

[37] Ghose T, Swendeman D, George S, Chowdhury D (2008). Mobilizing collective identity to reduce HIV risk among sex workers in Sonagachi, India: the boundaries, consciousness, negotiation framework. Soc Sci Med.

[38] van Zomeren M, Postmes T, Spears R (2008). Toward an integrative social identity model of collective action: a quantitative research synthesis of three socio-psychological perspectives. Psychol Bull.

[39] Ichoku HE, Ifelunini AI (2017). The changing political undercurrents in health services delivery in sub-Saharan Africa. Int J Health Serv.

[40] Fox AM, Reich MR (2015). The politics of universal health coverage in low- and middle-income countries: a framework for evaluation and action. J Health Polit Policy Law.

[41] Baum F, Freeman T, Sanders D, Labonté R, Lawless A, Javanparast S (2016). Comprehensive primary health care under neo-liberalism in Australia. Soc Sci Med.

[42] The Global Fund. Project Last Mile: The Coca-Cola Company. https://www.coca-colacompany.com/project-last-mile. Published 2019.

[43] Winters N, O’Donovan J, Geniets A (2018). A new era for community health in countries of low and middle income?. Lancet Glob Health.

[44] USAID and Center for Accelerating Innovation and Impact. Unleashing Private Capital for Global Health Innovation: Innovator and Investor Support Opportunities. https://issuu.com/usaidgh/docs/usaid_private_captial_508. Published 2019.

[45] Béland D (2016). Kingdon reconsidered: ideas, interests and institutions in comparative policy analysis. J Comp Policy Anal Res Pract.

[46] Baum FE, Sanders DM, Fisher M (2016). Assessing the health impact of transnational corporations: its importance and a framework. Global Health.

[47] Madureira Lima J, Galea S (2018). Corporate practices and health: a framework and mechanisms. Global Health.

[48] Schneider H, Maleka N (2018). Patterns of authorship on community health workers in low-and-middle-income countries: an analysis of publications (2012-2016). BMJ Glob Health.

[49] Richardson ET, McGinnis T, Frankfurter R (2019). Ebola and the narrative of mistrust. BMJ Glob Health.

[50] Maes K (2015). “Volunteers are not paid because they are priceless”: community health worker capacities and values in an AIDS treatment intervention in urban Ethiopia. Med Anthropol Q.

[51] McCollum R, Taegtmeyer M, Otiso L (2019). Applying an intersectionality lens to examine health for vulnerable individuals following devolution in Kenya. Int J Equity Health.

[52] Gaitonde R, Muraleedharan VR, San Sebastian M, Hurtig AK (2019). Accountability in the health system of Tamil Nadu, India: exploring its multiple meanings. Health Res Policy Syst.

